# Extensive Type A Aortic Arterial Dissection Presenting With Stroke Symptoms: A Case Report

**DOI:** 10.7759/cureus.55564

**Published:** 2024-03-05

**Authors:** Abdelrahman A Abdelhameed, Sumita Choudhary, Mohamed A Khoudir

**Affiliations:** 1 Stroke Department, Queen's Hospital, Romford, GBR

**Keywords:** dissection, stroke, acute type a aortic dissection, arterial dissection, aorta surgery

## Abstract

Aortic dissection (AD) is a rare but often lethal condition if not properly and urgently treated. Most often, patients arrive with acute hemodynamic instability and ripping chest agony. The patient’s life depends critically on a correct diagnosis made as soon as possible. We describe a 60-year-old man who arrived at the emergency room with symptoms of a brain stroke, including poor consciousness, left-sided weakness, and speech disturbance associated with hemodynamic instability, and chest pain. Thoracic aortic arch dissection was observed on CT angiography (CTA). In addition, CTA revealed that the dissection extends proximally into the left common carotid artery, left subclavian artery, brachiocephalic trunk, and right common carotid artery and distally to the left common iliac artery, coupled with significant stenosis of the left common iliac artery. Proper management of blood pressure (BP) parameters is life-saving for the patient. Since our hospital did not offer cardiothoracic surgery services, the patient was transferred to a different institution, where he received medical care immediately from an expert team and had surgery.

## Introduction

Aortic dissection (AD) is the disruption of the aorta's media layer, usually because of an intimal rupture. This causes bleeding in the aortic wall and causes the aorta's three layers to separate as a result [1]. With reported frequencies of five to 30 cases per one million individuals annually, AD is regarded as an uncommon event. AD usually manifests as acute hypotension accompanied by a ripping chest pain that radiates to the back. Nevertheless, if the carotid, vertebral, or spinal arteries are blocked, neurological manifestations may also occur. The most frequent neurological presentation is ischemic stroke, which frequently occurs with mild or without neck pain. Other neurological presentations include syncope, seizures, spinal cord ischemia, ischemic neuropathy, and encephalopathy [2]. We describe a patient who arrived at our stroke emergency department (ED) with acute chest pain, a left-sided face, arm and leg weakness, and slurred speech. Hemodynamic instability was shown to be the result of widespread type A acute AD, which caused symptoms of cerebral hypoperfusion presented with acute ischemic stroke symptoms.

## Case presentation

A 60-year-old right-handed gentleman with no history of past medical conditions and who is fully independent was blue-lighted to our hospital as a stroke call. He initially complained of chest discomfort to his wife, and then his consciousness level started to drop with the paramedical team in the ambulance, associated with a low blood pressure (BP) of 56/30 mm Hg. He presented to our hospital unconscious, with a Glasgow Coma Scale (GCS) of 7/15 of E2V1M4, left arm weakness, motor power 3/5, mild left facial droop, and dysarthria. It was difficult to assess the patient's language due to his low consciousness level, but he was deemed to have both receptive and expressive dysplasia due to generalized cerebral hypoperfusion. The total National Institutes of Health Stroke Scale (NIHSS) was 8/42. We were unable to assess his vision, sensation, or ataxia signs due to his low consciousness level and speech problems. There were two alarming clinical signs for the stroke team to think about a possible AD, which were chest pain with epigastric tenderness and low BP.

CT and CT angiograms (CTAs) of the head, chest, and abdomen showed significant findings of a hypodense lumen within the aortic arch, likely dissection extending into the aortic branches and distally into the thoracic aorta; proximally, the left common carotid artery tapers off and is narrow, referring to possible dissection. The left internal carotid artery is patent from the true lumen of the dissection. The dissection extends into the left subclavian artery, and the left vertebral artery is occluded completely from its origin. The intracranial left vertebral artery is collateralized by the vertebrobasilar junction. The dissection extends into the brachiocephalic trunk and into the right common carotid artery with mild narrowing and is focal. The true lumen of the right common carotid artery opacifies distally, and the right internal carotid artery is patent from the true lumen. A mild irregularity of the right vertebral artery was observed at the patent distally.

A non-contrast CT head showed no evidence of acute intracranial hemorrhage, extra-axial collections, or large vessel territory acute infarction, and the circle of Willis was patent with no large or medium intracranial arterial occlusion. An emergency cardiothoracic surgery referral was mandated. All attempts were made to refer him as soon as possible. During their contact, the patient's BP started to drop to 56/30 mm hg. The ITU team shared with the team and started to resuscitate the patient with intravenous fluids and metaraminol with the aim of keeping the systolic blood pressure at 90-100 mm hg and the mean BP at 60-65 mm Hg. The anesthesia team was ready to accompany the patient for transfer. The patient was not thromolyzed due to an improvement in his NIHSS from 8 to 3/42. He was not a candidate for mechanical thrombectomy due to the absence of a large vessel occlusion.

**Figure 1:**
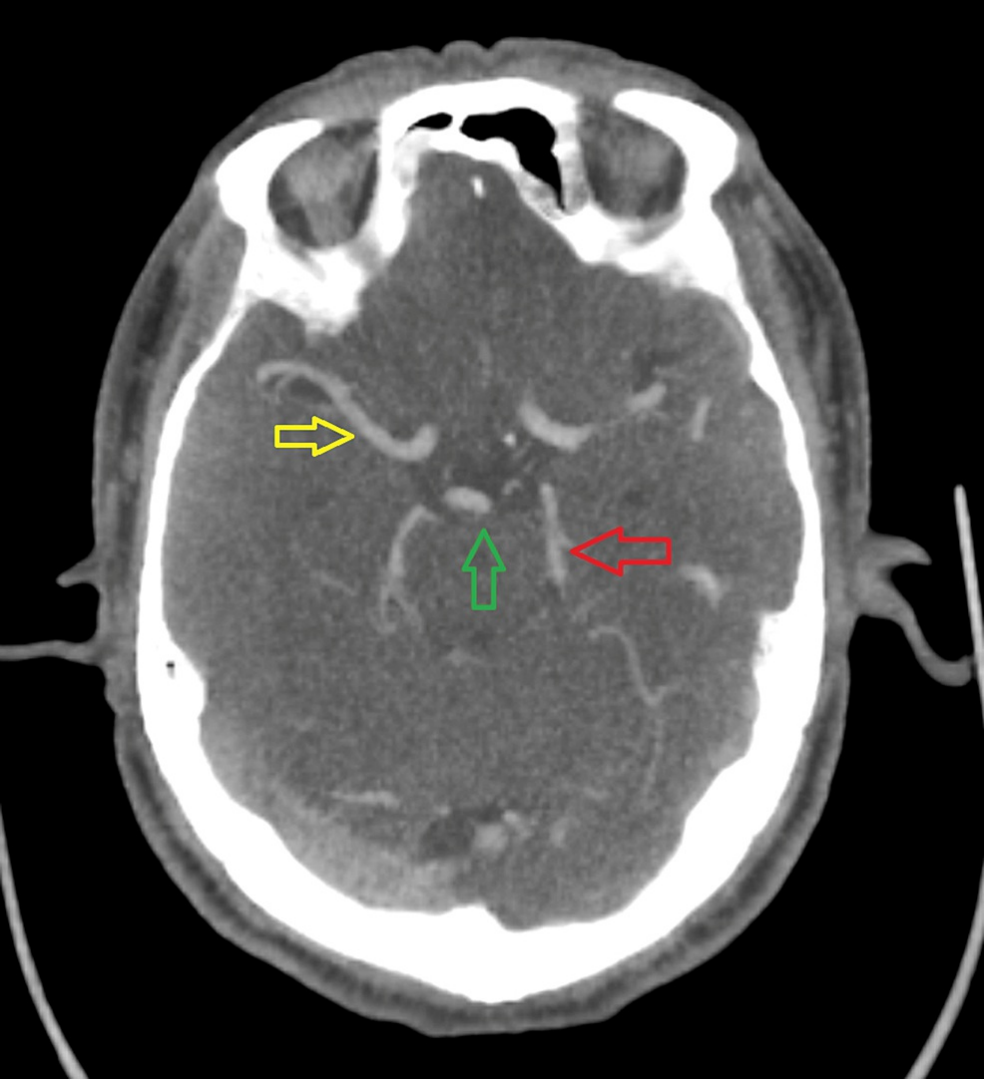
CTA of the brain showed patent anterior and posterior circulation. The yellow arrow points to the right middle cerebral artery. The red arrow points to the left posterior cerebral artery. The green arrow points to the tip of the basilar artery.

**Figure 2:**
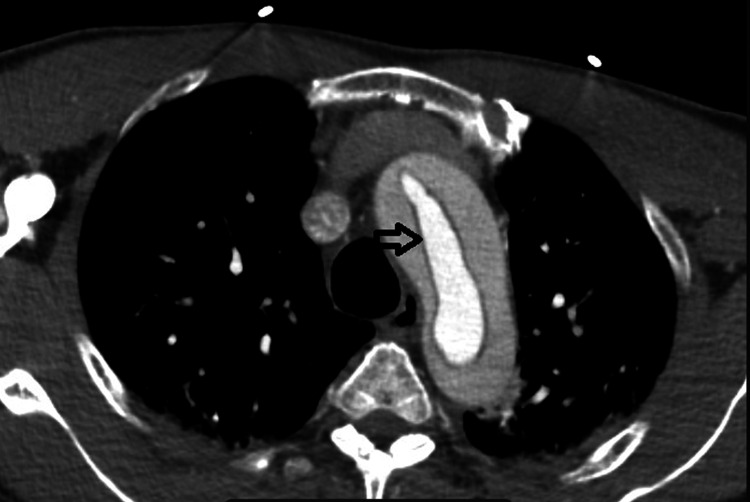
Axial CT angiography image at the level of the aortic arch showing a circumferential oval flap (windsock sign) involving the aortic arch (black arrow) results from intimo-intimal intussusception between the true and false dissected lumens.

**Figure 3:**
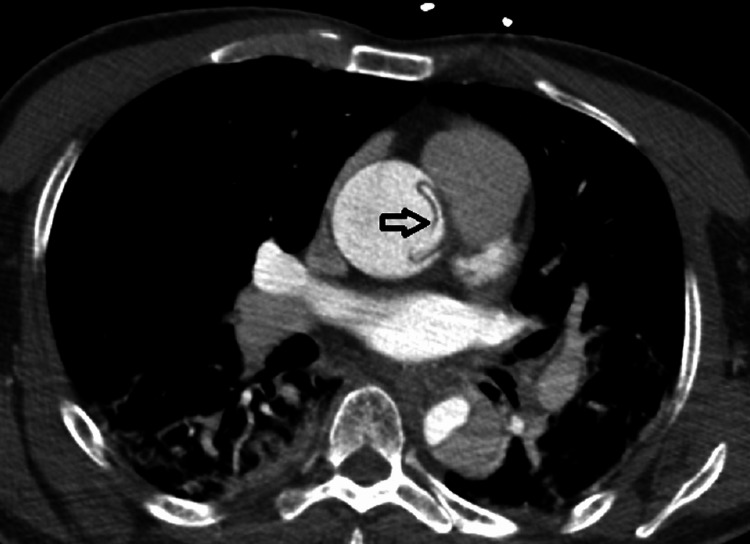
Axial CT angiography image of the thoracic region showing the dissection flap (black arrows) in the descending aorta.

**Figure 4:**
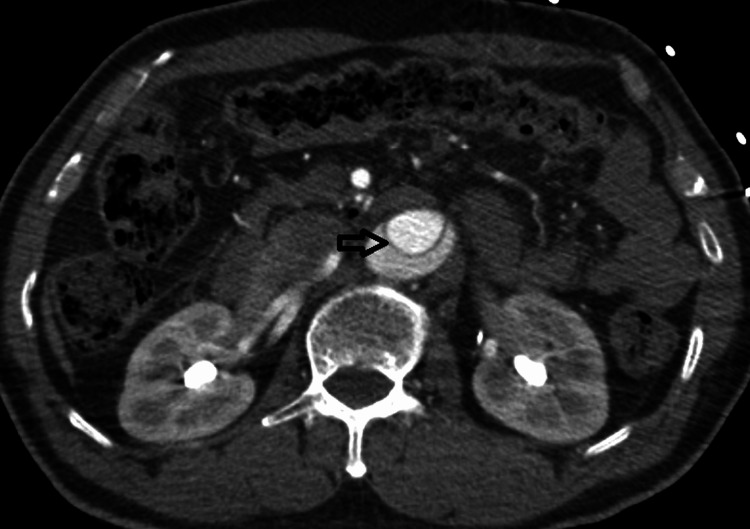
Axial CT angiography image of abdominal aorta showing linear hypodense structures within the false lumen (black arrow) with similar attenuation as the intimal flap (Cobweb sign).

During the arrangement for transfer, the patient had a CT aortogram, which showed a dissection flap (Figure 1) that began in the proximal aortic arch, extending into the descending thoracic aorta, abdominal aorta, and proximal left common iliac artery, associated with severe stenosis of the left common iliac artery, in which the dissection flap extended into. There was moderately severe stenosis of the true lumen of the thoracic and proximal abdominal aorta due to compression from the false lumen (Figure 2), but it remained patent. AD windsock (Figure 3) and Cobweb signs (Figure 4) were clearly illustrated.

**Figure 5:**
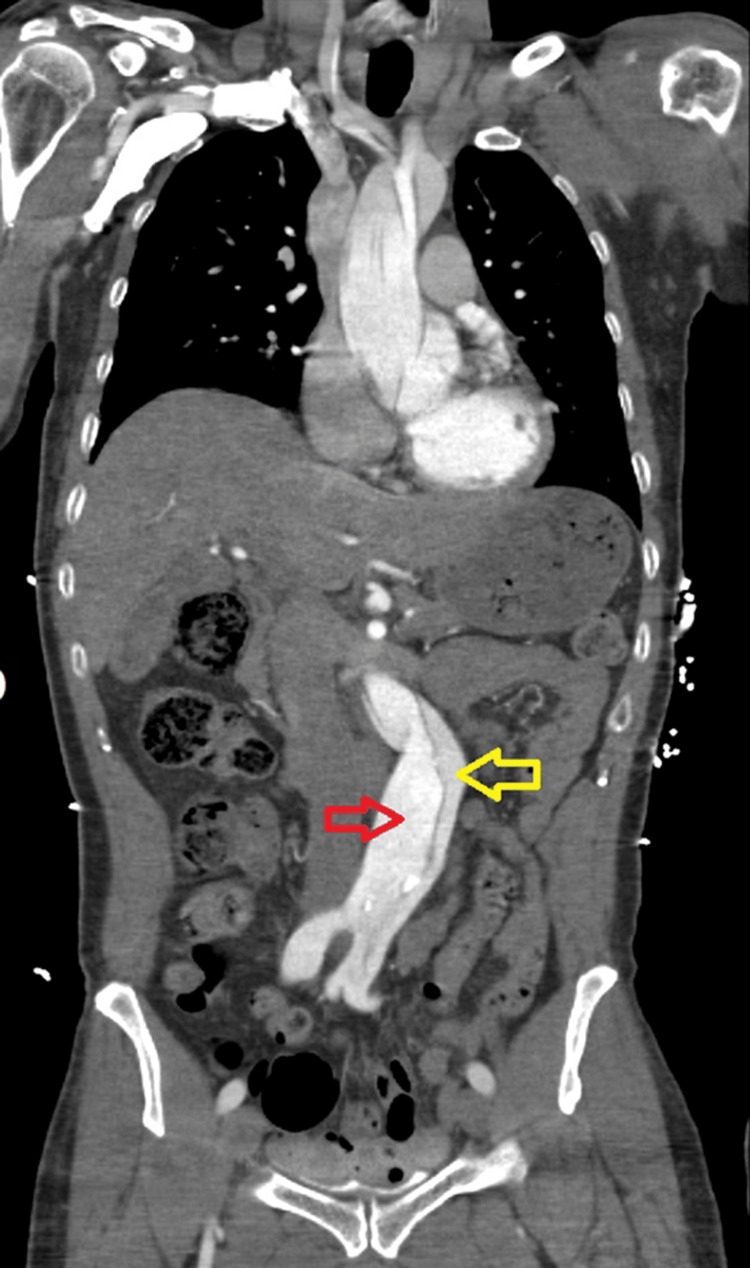
Coronal CT angiography image of the abdominal and thoracic aorta showing a true (red arrow) and false lumen (yellow arrow) with stenosis of the true lumen of the abdominal aorta due to compression from the false one.

**Figure 6:**
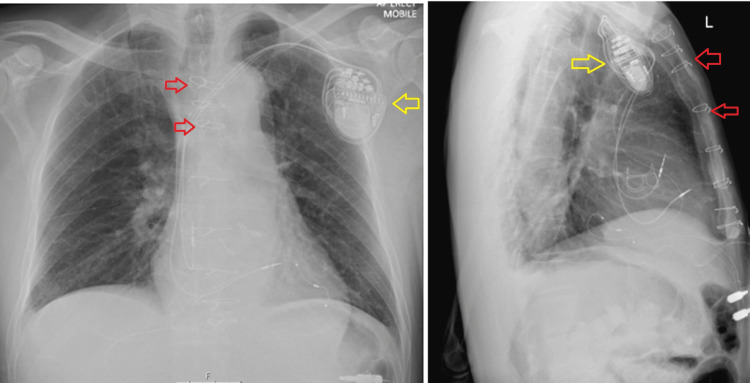
Postoperative X-ray showed thoracotomy suture (red arrows) performed to repair the aortic dissection with the permanent pacemaker inserted (yellow arrows).

The decision was made by the surgical team to perform emergency type A AD repair with tissue aortic root and ascending aortic and hemi-arch replacements. The entry tear was found in the mid-ascending aorta extending transversely across half of the circumference of the posterior aspect (Figure 5). No further tears were seen either in the aortic root or the aortic arch. Postoperatively, a slow second-degree heart block was used, upon which a permanent pacemaker was inserted (Figure 6). The patient was discharged after two weeks conscious, oriented, and alert. No MRI of the brain was done as the patient has made a full neurological recovery.

## Discussion

Early clinical picture of AD

The prevalence of AD presenting with neurologic symptoms in patients with AD is almost 37.7%. It may be challenging for medical professionals to diagnose AD due to the predominance of neurological symptoms at the time of the presentation [3]. Although chest discomfort is a well-known symptom of AD, it is not reported by up to one-third of individuals who come in with neurologic symptoms. Aphasia, forgetfulness, or a changed mental condition may complicate the initial assessment and obscure the way to rule out an accurate diagnosis of AD [4]. According to a 2018 meta-analysis on the history and exam features predictive of acute AD, focal neurologic deficits, pulse deficits, hypotension (BP < 90 mmHg), or symptoms of shock were the clinical indicators most indicative of acute AD and should alarm the examining physician to extensively investigate the aortic tree [5]. Our patient typically presented with chest pain followed by a low consciousness level and the development of neurological signs. His low BP urged the team to fully investigate the whole aortic vascular tree and confirm the diagnosis.

BP effect

The role of BP changes in suspecting an AD diagnosis is well documented in most of the literature. One of the most typical stroke presentations is hypertension. The systolic blood pressure (SBP) of 69% of individuals who suffered an immediate stroke was greater than 139 mmHg [6]. A similar outcome was noted in the research by Wallace et al., which showed that 84% of patients had a measured SBP greater than 150 mmHg [7]. In contrast to the analysis conducted by Shono et al., the mean diastolic BP (mean SD) and SBP of patients with neurological symptoms who had AD were 66.5 ± 17.3 mm Hg and 114.9 ± 28.9 mm Hg, respectively. There were no reports of severe hypertension [8]. In our instance, the BP was 56/30 mm Hg on initial presentation when a patient had neurological symptoms and an NIHSS of 8/42. Once the SBP optimized to 100/66 mmHg, the patient neurologically improved and had an NIHSS of 3/42. Imaging has revealed that this neurology is caused by low BP and inadequate brain perfusion as a result of AD.

Presentation of AD

With respect to the degree of involvement, AD can manifest in many ways. An acute onset of discomfort in the back, abdomen, or chest is usually seen in cases of AD. Type A ascending ADs are more likely to cause chest discomfort, whereas type B descending ADs are more likely to cause back and stomach pain [9]. AD can sometimes show painlessly. Patients who appear comfortably painless typically have a history of heart surgery, diabetes, or aortic aneurysm. Heart murmurs, pulse deficits, and variations in BP are examples of physical findings connected to the dissection. It is possible to observe focal neurological impairments with a thorough dissection that extends to the branch arteries [10].

Cardiac arrest and death can be the initial presentation of cases with AD as reported by Azhar et al. reported in his case report [11]. A patient presented with sudden collapse and weakness on the right side of her body. She was trying, but she went into cardiac arrest and passed away. The cause of death, as determined by the post-mortem examination, was hemopericardium caused by dissection of the ascending thoracic aorta. Two further case reports have mentioned syncope as the initial presenting condition. A 2017 case report details a type A AD dissection in a patient who initially presented with syncope and sporadic convulsions. Before collapsing, the patient was able to provide a prodromal history [12]. Syncope is defined as the first manifestation of a 49-year-old male with a history of hypertension who was later diagnosed with Stanford type A AD and had a poor clinical prognosis [13]. This case report was also published in the literature in 2021. Our understanding of the pathogenesis of acute AD has improved. The patient had initially reported the chest discomfort as tearing; however, as the dissection was being carried out, the pain subsided and was probably not entirely occluded by the intimal flap. We may believe that the chest discomfort was muted in part by the hypoperfusion to the brain hemisphere due to low BP and pain modulators.

Classification of AD

AD is divided into type A and type B according to the Stanford method. The ascending aorta and/or aortic arch are involved in type A. Type B begins distal to the left subclavian artery and affects the descending aorta. This case study presents an instance of type A AD involving the ascending thoracic aorta. Acute type A AD mortality risk is 0.5% per hour and 23.7% in 48 hours [14].

Diagnosis of AD

Transesophageal echocardiography, magnetic resonance imaging, and CT and CTA are the imaging modalities utilized to identify AD; CTA is typically the first modality of choice. Dissection of the aorta is suggested by the formation of a false lumen and the intimal flap that divides it from the actual lumen. Transesophageal echocardiography is usually recommended for patients who are hemodynamically unstable. When noninvasive modalities are equivocal and there is a high suspicion of AD, digital subtraction aortography is typically the preferred course of action [15].

It is frequently quite difficult to determine whether to use a surgical, endovascular, or conservative strategy for AD management. BP therapies, with or without antiplatelet therapy, and regular monitoring for ischemic neurological consequences would be an appropriate course of action in neurologically symptom-free individuals [16]. When a patient experiences multiple transient ischemic attacks (TIAs), symptomatic subclavian steal syndrome, or carotid hypoperfusion symptoms despite taking antithrombotic medication or having positive micro-embolic signal (MES) counts during transcranial Doppler (TCD) tests, surgery is indicated [17]. In our case, surgery was selected as the first option for intervention due to severe dissection involving multiple levels of the aorta, including the arch and the descending vessels with extensive intimal intussusception.

Role of BP control

Another therapeutic challenge in AD patients with acute strokes is managing BP. The American Heart Association (AHA) and American Stroke Association (ASA) suggest in their most recent guidelines establishing goals of "heart rate less than 60 bpm and an SBP between 100 and 120 mmHg" [1]. The most recent AHA/ASA guidelines for BP management in acute ischemic stroke advise patients who are candidates for tissue plasminogen activator (tPA) or mechanical thrombectomy to avoid hypotension and to maintain BPs less than 185/110 mmHg [18]. It is recommended that individuals who have ischemic stroke and AD should discuss their BP objectives with cardiothoracic surgery and neurology experts in the ED.

## Conclusions

AD is a serious disease that can present with symptoms of ischemic stroke of the brain. Physicians should maintain a high index of suspicion to consider AD for any patient who presents with focal neurologic symptoms, particularly if they are associated with chest pain, hypotension, or syncope. A full CTA of the brain and aortic tree is of paramount diagnostic value and is considered the modality of choice for guiding both the stroke and the cardiothoracic team for further management. Optimal BP parameters are still in favor of keeping an SBP of 100 mmHg to avoid further AD and total obstruction of the aortic lumen, together with maintaining brain perfusion. A specific level of BP control is still debatable in terms of the presence of neurological signs.
